# Mental Rotation of Digitally-Rendered Haptic Objects by the Visually-Impaired

**DOI:** 10.3389/fnins.2020.00197

**Published:** 2020-03-20

**Authors:** Ruxandra I. Tivadar, Cédrick Chappaz, Fatima Anaflous, Jean Roche, Micah M. Murray

**Affiliations:** ^1^The LINE (Laboratory for Investigative Neurophysiology), Department of Radiology, University Hospital Center and University of Lausanne, Lausanne, Switzerland; ^2^Department of Ophthalmology, University of Lausanne and Fondation Asile des Aveugles, Lausanne, Switzerland; ^3^Hap2u, Saint-Martin-d'Hères, France; ^4^Sensory, Perceptual and Cognitive Neuroscience Section, Center for Biomedical Imaging (CIBM), Lausanne, Switzerland; ^5^Department of Hearing and Speech Sciences, Vanderbilt University, Nashville, TN, United States

**Keywords:** blind, low vision, sensory substitution, mental rotation, haptics, digital technology

## Abstract

In the event of visual impairment or blindness, information from other intact senses can be used as substitutes to retrain (and *in extremis* replace) visual functions. Abilities including reading, mental representation of objects and spatial navigation can be performed using tactile information. Current technologies can convey a restricted library of stimuli, either because they depend on real objects or renderings with low resolution layouts. Digital haptic technologies can overcome such limitations. The applicability of this technology was previously demonstrated in sighted participants. Here, we reasoned that visually-impaired and blind participants can create mental representations of letters presented haptically in normal and mirror-reversed form without the use of any visual information, and mentally manipulate such representations. Visually-impaired and blind volunteers were blindfolded and trained on the haptic tablet with two letters (either L and P or F and G). During testing, they haptically explored on any trial one of the four letters presented at 0°, 90°, 180°, or 270° rotation from upright and indicated if the letter was either in a normal or mirror-reversed form. Rotation angle impacted performance; greater deviation from 0° resulted in greater impairment for trained and untrained normal letters, consistent with mental rotation of these haptically-rendered objects. Performance was also generally less accurate with mirror-reversed stimuli, which was not affected by rotation angle. Our findings demonstrate, for the first time, the suitability of a digital haptic technology in the blind and visually-impaired. Classic devices remain limited in their accessibility and in the flexibility of their applications. We show that mental representations can be generated and manipulated using digital haptic technology. This technology may thus offer an innovative solution to the mitigation of impairments in the visually-impaired, and to the training of skills dependent on mental representations and their spatial manipulation.

## Introduction

A major issue for the visually-impaired is mobility. Visually-impaired and blind individuals have higher risks of unintentional injuries, both at home and in the general environment (Legood et al., 2002; Manduchi and Kurniawan, 2011). Mobility depends on the integrity of our spatial functions, which in turn depend on mental representations that themselves rely on the correct functioning of cortical visual mechanisms (Thinus-Blanc and Gaunet, 1997). Loss of visual functions through visual impairment or blindness can affect the way that mental representations are created, which in turn leads to impairment in multiple functions such as reading, manipulation of objects, or orientation in space (Thinus-Blanc and Gaunet, 1997; Kuyk et al., 2004; Lahav et al., 2012). In fact, visual impairments affect nearly 300 million people globally, with another ~36 million suffering from complete loss of vision (World Health Organization, 2000). Therefore, studying how mental representations can be established and manipulated in visually-impaired and blind individuals is arguably an important public health issue.

Spatial functions can be supported by visual, tactile, and auditory stimuli (Auvray et al., 2007; Collignon et al., 2007; Lacey et al., 2007a; see Lacey and Sathian, 2012, for a review). Specifically, studies of tactile mental rotation in the blind demonstrate a typical decrease in performance with increasing image rotation (Shepard and Metzler, 1971; Marmor and Zaback, 1976; Prather and Sathian, 2002), consistent with a classic mental rotation effect (Shepard and Metzler, 1971) as found for visual stimuli (Jordan et al., 2001; Thomas et al., 2013; Iachini et al., 2019). This has led to research demonstrating that spatial representations can be achieved in a largely modality-independent fashion (Lacey and Campbell, 2006), engaging a common representational system (Lacey and Sathian, 2012; Masson et al., 2016). The ability of visually-impaired individuals to use tactile information to analyze spatial properties, as well as the modality-independence of spatial skills, have opened new avenues for rehabilitation via sensory substitution. Multiple forms of tactile sensory substitution devices (SSDs), such as the classic Braille alphabet and white cane, but also more novel devices such as the Tongue Display Unit (TDU) (Chebat et al., 2007), focus on rehabilitating functions such as reading and orientation in space. However, such SSDs risk remaining limited in their applications, due to limited libraries of stimuli, persistent training, and restricted accessibility/ergonomics. Moreover, devices such as the TDU or BrainPort (Bach-y-Rita et al., 2005; Arnoldussen and Fletcher, 2012) are invasive (Gori et al., 2016), in that they block the tongue, and that they deliver voltage impulses which, on the long run, might negatively affect the skin through repeated stimulation (Fary and Briffa, 2011), potentially leading to tissue damage and pain (Prausnitz, 1996). New non-invasive applications aiming to digitally render tactile information promise to resolve such issues through digitalization. This digitization of information has led to significant improvements in healthcare, including reduced costs and increased accessibility and reliability of treatments (Noffsinger and Chin, 2000; Dwivedi et al., 2002). Currently, visually-impaired individuals require many training hours together with occupational therapists in order to rehabilitate visual functions such as reading or orientation in space. By contrast, digitizing the delivery method of such therapies would reduce resources for the medical domain, as well as increase patient independence. Thus, patients could train both their tactile perception, as well as their shape and space perception, without the constant supervision of a therapist. In addition, the non-invasive nature of a technology based on ultrasonic vibrations rather than electrical stimulation, such as the one employed in our study does not entail the possibility of negative long-term effects such as those induced by prolonged electrical stimulation.

It has previously been shown that sighted participants can use digital haptics to create and manipulate mental representations of letters (Tivadar et al., 2019). In this study, Tivadar and colleagues tested a group of sighted subjects on a mental rotation task with digitally-rendered haptic stimuli on the same prototype as in the current study. The authors had participants actively explore haptic letters that were simulated on the screen of the tablet, in order to recognize whether these letters were presented in normal or mirrored forms. Critically, these letters could be rotated at four angles (0°, 90°, 180°, 270°), thus obligating participants to engage in mental rotation of the presented stimuli. (Tivadar et al., 2019) results support the fact that participants successfully managed to build mental representations of these digitally-presented haptic letters, that they were then able to rotate so as to correctly determine the form. It has also been shown that mental rotation in the visually-impaired can be supported by tactile stimuli (see Prather and Sathian, 2002, for a review). However, it remains unknown whether visually-impaired and blind individuals can use simulated digital tactile information to build mental representations of objects that they can then also mentally manipulate. In fact, if these digital applications are well-suited to rehabilitate spatial functions, this would highly increase the speed of recovery of such patients. As such, these applications are very promising due to the ease of delivery of digital information, their independence of environment, and the fact that they can easily simulate real-world objects and environments. This in turn supports everyday functions such as localization, mapping, and building internal representations of objects, thus entailing a high translational facility to veridical environments. In addition, being able to create and rotate mental representations of objects based on digital simulated haptic information is an important step in a proof-of-concept for the successful acquisition and manipulation of a simulated haptic space.

In order to investigate whether visually-impaired and blind participants would be able to successfully create and manipulate mental representations of objects, we tested a group of subjects suffering from visual impairments of different types and severities on a mental rotation task, using normal and mirror-reversed digital haptic letters. We chose to have a heterogeneous group in terms of their visual impairment, due to the fact that such applications are aimed at individuals living with various forms of visual impairment. We hypothesized that visually-impaired and blind individuals should show the classic mental rotation effect, as first investigated by Shepard and Metzler (1971), meaning decreasing performance with increasing object rotation. If so, this would mean that participants are able to use digital haptic information to create mental images of 2-D objects, such as letters. Specifically, we expected to see this effect for normal trained as well as new stimuli, contrary to results in sighted (Tivadar et al., 2019), where sighted participants did not perform well with untrained (new) letters. We expect this given higher tactual expertise of visually-impaired and blind participants (Goldreich and Kanics, 2003; Legge et al., 2008; Wong et al., 2011). Similar, yet worse, performance is expected for mirror-reversed stimuli as compared to stimuli presented in their normal form, given previous evidence demonstrating slower reaction times and higher errors with stimuli in their mirrored as compared to normal form (Marmor and Zaback, 1976; Carpenter and Eisenberg, 1978), and the stimulus familiarity effect (White, 1980; Bethell-fox and Shepard, 1988).

## Methods

### Participants

Written informed consent was obtained from each participant to procedures approved by the cantonal ethics committee. Fourteen adults (7 women and 7 men; age range 18–64 years, mean ± SD: 40 ± 12.6 years) were tested. Each was compensated 50 Swiss francs for their participation. Ten of the participants were completely blind, and 4 retained some low vision. Most participants were right-handed, only one was left-handed. Regarding Braille literacy, six of them reported good literacy, six of them reported no literacy, and two of them reported little literacy. Most of our participants had an acquired visual impairment or blindness (*N* = 10), and four of them were congenitally blind. Diagnostic visual acuity measurements were transformed into logMAR (Bonavolonta et al., 2010; Patel et al., 2017). Characteristics about patients' demographics and diagnoses are presented in Table 1. No participant reported tactile deficits or had a history of or a current neurological or psychiatric illness.

**Table 1:** Patient characteristics and group assignment.

**ID**	**Gender**	**Age range**	**Diagnosis**	**Congenital /acquired**	**Years of visual impairment**	**Vision**	**Residual vision**	**Visual acuity [logMAR] (right eye; left eye)**	**Braille**	**Locomotion**	**Group of letters trained**	**Inclusion in analyses**
Pat06	F	55–60	Retinitis Pigmentosa & deafness (corrected)	Acquired	14	Blindness	Right eye: 0.03, left eye: 0.03	(1.5; 1.5)	Yes	Cane	2	Y
Pat01	M	60–65	Retinal Vascular Occlusion (L&R)	Acquired	7	Blindness	Total blindness	(3; 3)	Little	Dog	1	Y
Pat15	F	40–45	Congenital retinopathy, both eyes enucleated	Congenital	44	Blindness	Total blindness	(3; 3)	Yes	Cane	1	Y
Pat02	M	25–30	Glaucoma	Acquired	13	Low vision	Visual field <15°, right eye: 0.63, left eye: 0.05	(0.2; 1.3)	No	cane	2	Y
Pat11	F	40–45	Usher Syndrome: Retinitis Pigmentosa, deafness (corrected)	Acquired	7	Low vision	Right eye: 0.16, left eye: 0.16	(0.8; 0.8)	No	Cane	1	Y
Pat14	F	35–40	Optic Nerv Atrophy	Congenital	38	Blindness	Right eye: luminous perception, left eye: hand movements	(2.9; 2.9)	Yes	Cane	2	Y
Pat12	F	40–45	Retinitis Pigmentosa	Acquired	36	Blindness	Luminous perception, shapes	(2.9; 2.9)	No	–	2	Y
Pat13	M	30–35	Optic Nerv Atrophy	Acquired	3	Blindness	Right eye: perception of hand movements, left eye: counts fingers at 1.5 m distance	(2.3; 1.7)	Yes	Cane	2	Y
Pat04	F	30–35	Congenital Glaucoma	Congenital	33	Blindness	Right eye: luminous perception, left eye: total blindness	(2.9; 3)	Yes	Dog	1	Y
Pat09	M	25–30	Lyell syndrome	Acquired	15	Blindness	Right eye: 0.05, left eye: luminous perception	(1.3; 2.9)	No	Cane	1	Y
Pat10	M	25–30	Leber congenital Amaurosis	Acquired	6	Blindness	Right eye: 0.05, left eye: < 0.05	(1.3; <1.4)	Little	Independent	2	N
Pat03	F	55–60	Divergent strabismus, macular hole, cataract (R)	Acquired	6	Low vision	Right eye: 0.2	NA	–	Cane	2	N
Pat16	M	18–20	Leber congenital Amaurosis	Congenital	19	Blindness	Luminous perception	(2.9; 2.9)	Yes	Cane	1	N
Pat08	M	30–35	Usher Syndrome: Retinitis Pigmentosa, deafness (corrected)	Acquired	13	Low vision	Right eye: 0.25; left eye: 0.4	(1.6; 0.4)	No	Independent	1	N

*M stands for Male, F stands for female. Training 1 stands for training on the letters L and P, and Training 2 stands for training on the letters F and G*.

### Apparatus

The apparatus was identical to that used in Tivadar et al. (2019). It entailed a 7″ tablet with a 1,024 × 600 pixel touchscreen. The tablet's operating system is Linux-based (Raspbian) and the tablet itself is equipped with a software tool to render and control the presentation of haptic textures. Briefly, the software recodes images in jpeg format into a haptic format using a kit written in C++. Full details of the technique and the haptic rendering are provided in Vezzoli et al. (2016, 2017) and Rekik et al. (2017).

### Stimuli

**Figure 1:**
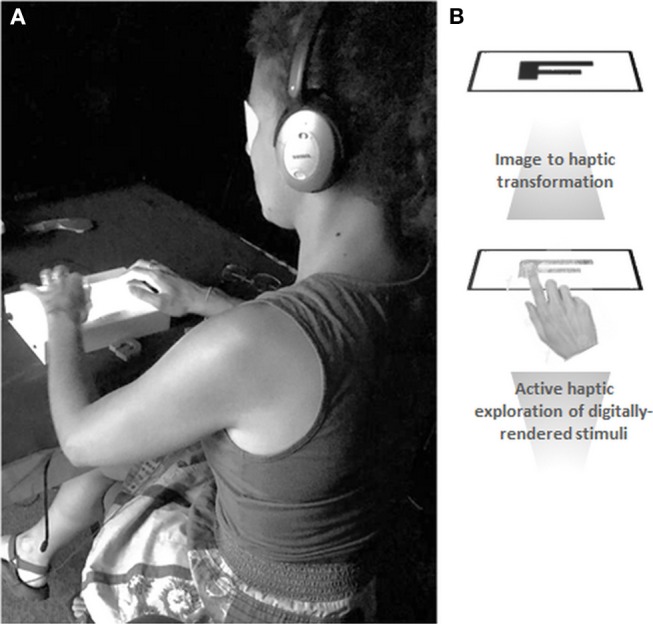
Experimental setup and digital haptic transformation. **(A)** Each subject wore a blindfold and noise-canceling earphones throughout the experiment. This was done to prevent any residual or inadvertent visual input and to mask any audible noise from the tablet. During each trial, the subject could explore the letter stimulus for 30 s with one finger of their dominant hand. They then used their non-dominant hand to respond via a computer mouse whether the letter was upright or mirror-reversed. **(B)** The tablet's pre-installed software development kit translated the image files into haptic renderings. The transformation involves delineating the image in a series of 8 × 8 pixel cells and coding each of the cells into textures, using a haptic library where different textures are defined. Written informed consent was obtained from the individual for the publication of this image.

Stimuli were identical to those employed in Tivadar et al. (2019). These were 4 upper-case letters—L, P, F, and G—drawn in Paint (see e.g., Carpenter and Eisenberg, 1978; see also Figure 1). We also used Matlab to generate images wherein each letter was rotated 0°, 90°, 180°, or 270° from upright and mirrored. In terms of the conversion from image to haptic rendering, the stimuli appeared centrally and on a white (i.e., blank in terms of haptic rendering) background. All pixels of the letter stimuli were coded with the same haptic texture, based on the Hap2u pre-installed Texture Editor software. The shape of the ultrasonic vibration was a square wave of roughly 2 μm amplitude (see e.g., Sednaoui et al., 2017). This has been shown to produce the most intense and quick reduction of the friction of the screen under the finger; i.e., a “pointy” sensation. Additionally, a “coarse” texture was produced by using a 3,500 μm period of the square wave (see Hollins and Risner, 2000).

### Procedure and Task

Likewise, the procedure and task are identical to what is described in Tivadar et al. (2019). All participants completed the experiment within a sound-attenuated, darkened room (WhisperRoom MDL 102126E). All of our participants wore a blindfold as well as noise-canceling headphones (Bose model QuietComfort 2). This was done so as to block any potential residual light and to mask any sounds from the tablet (see also Murray et al., 2005). This may likely minimize any cross-modal facilitation (Lacey et al., 2007b), impeding a recognition of the presence/absence or location of the letters by the sound the tablet made when explored. We would note that while all participants were familiar with letters, no participant was familiar with the haptic tablet or had prior exposure to the haptic stimuli. We instructed participants to explore the tablet with a finger from their dominant hand and to respond with their non-dominant hand. For each letter, the participant was asked to indicate via a computer mouse if it was presented in normal or mirror-reversed manner (left and right buttons, respectively). We explicitly instructed participants to explore the haptic rendering, to identify the letter, and to imagine rotating the letter to upright (0°) so as to judge if the letter was normal vs. mirror-reversed. Stimulus duration was 30 s followed by a 20 second response window. These timings were determined during pilot experiments, where it was evident that participants needed some time to recognize when a stimulus appeared on or disappeared off the screen of the tablet. The ensuing trial was initiated after the response and with the further inclusion of an inter-trial interval ranging 500–1,000 ms. To train the participants with the haptic tablet, we had each of them complete three training blocks, each comprised of 16 trials (2 per condition). During training, a participant was exposed to only 2 of the 4 letters (either L and P or alternatively only F and G). We counterbalanced across individuals the letters to which they were exposed. The pairing of letters was based on their perceptual proximity, which allowed a progressive learning procedure. We trained participants with pairs of letters in this way in order to assess whether effects generalized to untrained stimuli. Prior to exposure to the letter stimuli, we first trained participants to explore the tablet screen via lateral sweeps (Stilla and Sathian, 2008). While we did allow participants to change the finger used for exploring the tablet (to minimize any adaptation or habituation effects), we did not allow for changing hands. We then taught participants to differentiate vertical from horizontal lines. Afterwards, participants completed the abovementioned training blocks. The experimenter (R.I.T.) provided participants with verbal instructions as well as feedback regarding general performance and the accuracy of responses throughout the training session. The testing phase comprised 3 blocks of 32 trials, making 96 trials in total per participant [i.e., six trials per each of the 16 combinations of 4 angles (0°, 90°, 180°, and 270°) × 2 conditions (normal/mirror) × 2 training (trained/untrained)]. During the experiment, participants were ecnouraged to take regular breaks between blocks of trials to maintain high concentration and prevent fatigue. The total experiment duration was no longer than 3 h 30 min. Stimulus delivery and behavioral response collection were controlled by Psychopy software (Peirce, 2007).

### Behavioral Analysis

**Figure 2:**
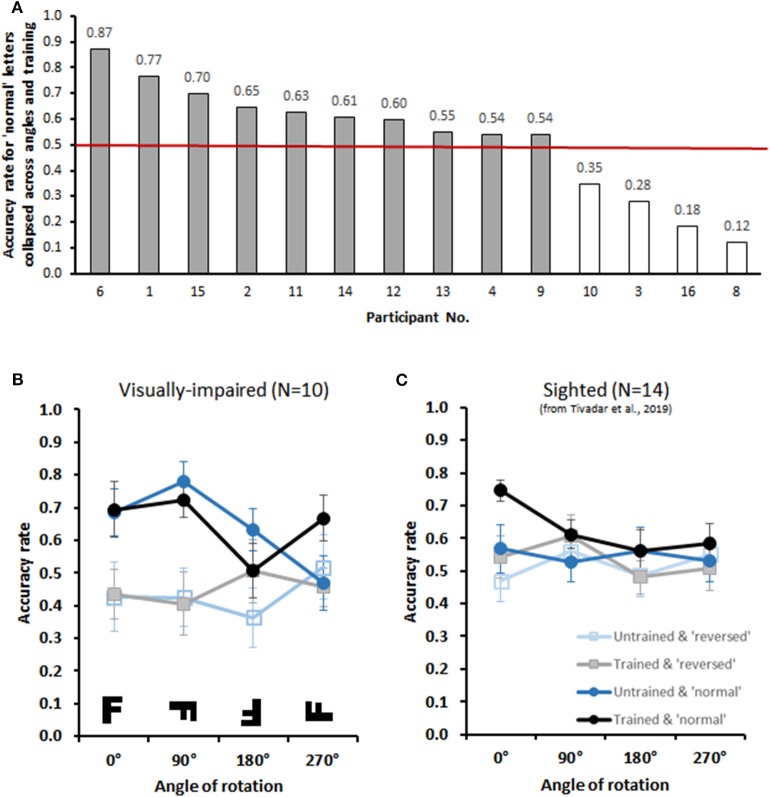
Behavioral Results **(A)** The bar graph displays individual performance (accuracy rates) with trained normal letters collapsed across all angles. The red line indicates chance performance (i.e., 0.50). Participants were included if performance was above chance, resulting in the exclusion of 4 participants. **(B)** The lower graphs display group-averaged performance for upright trained, upright untrained, reversed trained, and revered untrained letters at four orientations (visual versions displayed in the inset) for the group of 10 included participants. There was an archetypical mental rotation effect with upright letters, independently of whether or not they were trained. This was not the case for mirror-reversed letters. **(C)** Comparative data from sighted individuals performing the identical task as originally reported in Tivadar et al. (2019).

We used Matlab to pre-process the data and R (R Core Team, 2018) for analyses. First, we excluded all trials that were classed as missed trials, i.e., trials with RTs over 20 s (15.1% of trials). Any remaining outlier trials were then excluded on a single-subject basis (i.e., for each subject and condition), applying a criterion of the mean ±2 standard deviations around their RTs (2.9% of all trials, see Ratcliff, 1993; Field, 2012). Accuracy means were calculated from the remaining trials. Missing means in certain conditions were replaced with the mean of all subjects for the specific condition (14 cases in total, 8.75% of total data). Upon inspection of Accuracy scores, we found 4 of the 14 patients had a global performance (i.e., across all angles of presentation) for letters in their normal form that was equal to or lower than chance, i.e., ≤50% (Figure 2A). We excluded these participants. Our final group comprised 10 participants (aged 27–64 years; mean ± SD: 40.7 ± 12.1 years). We compared Accuracy with a 2 × 2 × 4 repeated measures ANOVA with factors Training (trained/untrained), Condition (normal/mirror) and Angle (0°, 90°, 180°, 270°), after not having found a significant deviation from the normal distribution and from homoscedasticity (assumptions tested with Shapiro and Levene tests). Greenhouse-Geiser corrections were applied whenever sphericity was violated. Partial eta-squared is reported as a measure of effect size. As in Tivadar et al. (2019), RTs were not further analyzed as they represented somewhat cued responses, and were not deemed informative as such. More specifically and as described above in the Methods section, participants had to explore the tablet for 30 s before being able to give responses on any given trial. This was done out of practical reasons, in order to ensure correct interaction with the tablet. Therefore, RTs are not representative of the time it took participants to correctly identify the form of a given letter, as even if participants had correctly identified the letter before the 30 s passed, they were not able to respond.

## Results

### Accuracy

Group-averaged (*N* = 10) accuracy rates are displayed in Figure 2B. The 2 × 2 × 4 repeated measures ANOVAs (Training × Condition × Angle) exhibited a significant 3-way interaction (Training × Condition × Angle) [*F*_(3,27)_ = 3.28, *p* = 0.04, η_*p*_^2^ = 0.27], and a significant 2-way interaction (Condition × Angle) [*F*_(3,27)_ = 3.01, *p* = 0.05, η_*p*_^2^ = 0.25]. There was also a main effect of Condition [*F*_(1,9)_ = 7.6, *p* = 0.02, η_*p*_^2^ = 0.46], with generally higher accuracy on Normal than on Mirrored letters. We therefore next conducted two separate 2 × 4 repeated measures ANOVAs (Trained × Angle) for Normal and Mirrored letters. For Normal letters, we observed a significant Trained × Angle interaction [*F*_(3,27)_ = 3.81, *p* = 0.02, η_*p*_^2^ = 0.30]. Despite this interaction, the *post-hoc* contrasts comparing accuracy rates for each angle revealed no significant differences between Trained and Untrained conditions (all *p*′s > 0.08; without correction for multiple comparisons). Crucially, however, the within-subjects contrasts revealed a linear main effect of Angle [*F*_(1,9)_ = 10.00, *p* = 0.01, η_*p*_^2^ = 0.53]. Participants thus demonstrated typical mental rotation effect (Shepard and Metzler, 1971), i.e., decreasing performance accuracy with increasing angular disparity from upright, for letters presented in their normal form. No significant interactions or main effects were observed for Mirrored letters (*F*′s < 0.9).

We have previously provided a proof-of-concept for sighted subjects with this same protocol as well (Tivadar et al., 2019). We display these data here in Figure 2C. In this prior work, we observed a mental rotation effect for trained letters, but not for untrained letters, presented in their normal format. Given that identical paradigms and equipment were used in both studies, we *a posteriori* compared performance across sighted and visually-impaired for the normal format condition, as these were those exhibiting a mental rotation in each group when studied separately. Specifically, we performed a 2 × 2 × 4 mixed model repeated measures ANOVA with group as the between-subjects factor and Trained and Angle as the within-subjects factors, as described above. The within-subjects contrasts revealed a significant 3-way interaction [*F*_(1,22)_ = 6.71, *p* = 0.017, $\eta_{p}^{2}$ = 0.23]. To better understand the basis for this interaction, additional 2-way mixed model ANOVAs were run for trained and untrained conditions separately. For trained letters, there was a linear main effect of angle [*F*_(1,22)_ = 6.89, *p* = 0.015, $\eta_{p}^{2}$ = 0.24], as expected, but no significant interaction with group (*p* > 0.45). By contrast, for untrained letters, there was both a linear main effect of angle [*F*_(1,22)_ = 9.99, *p* = 0.005, $\eta_{p}^{2}$ = 0.31], as expected, as well as a significant interaction with group [*F*_(1,22)_ = 6.93, *p* = 0.015, $\eta_{p}^{2}$ = 0.24]. This overall pattern indicates that visually impaired individuals exhibit greater generalization to untrained letters than their sighted counterparts (Figures 2B,C).

## Discussion

We provide the first proof-of-concept that visually-impaired and blind individuals were able to use digitally-rendered haptic letter stimuli to create mental representations that they could then spatially manipulate on a task requiring the discrimination of normal vs. mirror-reversed letters. In particular, patients showed the classic mental rotation effect (Shepard and Metzler, 1971), demonstrating decreasing performance with increasing angular disparity, or rotation of the letters, they previously haptically explored. This effect was visible not only for trained letters in their normal but not mirrored format, but also generalized to untrained letters. This is an important result, because it represents a necessary validation of the suitability of digital haptics for the rehabilitation of functions that rely on manipulable mental representations in the visually-impaired.

We furthermore compared the performance of our visually-impaired and blind participants with that of sighted individuals described in our prior published work (Tivadar et al., 2019). In this prior work, using the same device and protocol as here, we observed a mental rotation effect for trained, but not untrained, letters presented in their normal format. The results across the studies differed insofar as visually-impaired and blind individuals exhibited similar performance with both trained and untrained letters, whereas sighted participants showed no evidence for the generalization to untrained letters. It may be that vision (and individual's potential to use visual imagery) deleteriously affects the ability to generalize effects based on haptic-based mental representations from trained to untrained letters. Another alternative, which is not mutually exclusive, is that visually-impaired and blind individuals have greater tactile experience that in turn facilitates the observed generalization. Ample evidence indicates that tactile processing is enhanced by prolonged visual deprivation (Doane et al., 1959; Zubek et al., 1964; Facchini and Aglioti, 2003; Chebat et al., 2007; Wong et al., 2011). However, there is still debate regarding the extent to which short-term visual deprivation can enhance passive tactile discrimination. For example, Wong et al. (2011) observed no enhanced *passive* tactile grating orientation discrimination after short visual deprivation (up to 110 min) of their sighted subjects. By contrast, Facchini and Aglioti (2003), observed enhanced passive tactile grating orientation discrimination after 90 min of blindfolding, and Leon-Sarmiento et al. (2008) observed enhanced discrimination of grating orientations after only 45 min of visual deprivation. Thus, even if we do not assume that blindfolding our sighted participants enhanced their tactile acuity, we suggest, given the above evidence, that visual deprivation impacts tactile discrimination abilities including the ability to form and manipulate mental representations of objects (here letters).

### Implications for Models of Perceptual Encoding in the Blind and Visually-Impaired

The fact that visually-impaired and blind participants exhibited mental rotation effects for both trained and untrained normal stimuli might be indicative of an *item-independent* encoding system operating in these individuals, which allows for facilitated transfer between new and learnt stimuli and faster generalization of encoding rules. It was previously proposed that this might be a result of changes in stimulus processing and encoding that are driven by neuroplasticity (Collignon et al., 2006, 2009, 2015). Specifically, it was suggested that the congenitally blind may show different learning strategies from the sighted as a result of allocating more attention to sensory information processing (Pring, 1988; Collignon et al., 2006). In fact, to test the hypothesis that the lack of visual input results in data-driven rather than conceptual encoding strategies, Röder and Rösler (2003) tested memory for environmental sounds in sighted, congenitally-blind and late-blind subjects. They had participants encode sound either in a semantic (“deep”) or in a physical (“shallow”) fashion, and found that while all three groups profited most from semantic encoding, congenitally blind individuals outperformed the sighted ones on conceptually similar items after encoding (Röder and Rösler, 2003), and age-matched late-blind performed as well as congenitally-blind participants. Such results refute previous beliefs that the blind are less able to use conceptually based encoding strategies (for a discussion, see Thinus-Blanc and Gaunet, 1997), and instead support the hypothesis that the visually-impaired are able to make elevated use of perceptual encoding to aid recognition (Röder and Rösler, 2003; Rokem and Ahissar, 2009). Our results are also consistent with these hypotheses. The lack of differences in performance of our participants between trained and untrained stimuli suggests that once a general schema of stimulus encoding is created, visually-impaired and blind individuals easily transfer the learned concepts to unfamiliar stimuli. The visually-impaired and blind might thus rely more on conceptual item-independent processing (Röder et al., 1997; Collignon et al., 2006; Rokem and Ahissar, 2009) that may help compensate for visual loss.

### Implications for Rehabilitation via Sensory Substitution

The multisensory or “supra-modal” nature of object and spatial representations has important implications for rehabilitation applications using sensory substitution in individuals where input from one sensory modality, for example vision, is either impaired or absent. In such cases, both multisensory and cross-modal processes are primary drivers of neuroplasticity in visual areas (Kirkwood et al., 1996; Amedi et al., 2004; Merabet et al., 2005; Pascual-Leone et al., 2005; Murray et al., 2015), which may promote a task-selective and modality-independent re-specialization of these cortical structures (Murray et al., 2016; Amedi et al., 2017). In addition, spatial feature processing does not generally seem to rely on information from a specific modality (Pribram, 1971; Amedi et al., 2001; Pietrini et al., 2004; Struiksma et al., 2009). Lacey et al. (2007b) also demonstrated that such “supra-modal” representations of spatial characteristics are viewpoint-independent, and thus unaffected by object constancy issues (see also Lacey et al., 2009). One implication is that it may prove easier to achieve an “abstract” object representation (Pietrini et al., 2004). It has been repeatedly demonstrated that tactile information specifically can support spatial functions in blind, visually-impaired, and sighted subjects (Marmor and Zaback, 1976; Carpenter and Eisenberg, 1978; Grant et al., 2000; Ptito et al., 2005; Sathian, 2005; Chebat et al., 2007; Wan et al., 2010; Rovira et al., 2011; Vinter et al., 2012).

However, classical devices for conveying tactile information, such as the Braille alphabet, the white cane, and tactile maps, remain limited in the breadth of information they can convey, their accessibility, and their ergonomics (among other considerations) (reviewed in Gori et al., 2016). There are several potential advantages of digital haptics using the finger/hand over technologies such as the BrainPort or TDU delivered to the mouth. For one, digital haptics are completely non-invasive. The abovementioned technologies are not only ergonomically invasive (i.e., the mouth is full), but also use electrical stimulation of the tongue. Second, participants using the TDU needed 9 h of training on a single letter from Snellen's E test (i.e., the letter E) to recognize the letter presented in sizes ranging from 5 to 1.3 cm with 100% accuracy (see Figure 2 in Sampaio et al., 2001). By comparison, our participants needed 45 min to recognize trained and untrained letters in their normal form at a 0° and 90° angle (i.e., 4 letters, size on screen height × length: 4 × 5 cm) with ~70–75% accuracy. Thus, our method allows faster learning of a wider vocabulary of stimuli. In addition, the renderings via digital haptics allow for rapid and even dynamically changing simulation of a wide variety stimuli (from letters to full spatial maps) that need only to be digitally translated (i.e., coded) into a haptic form. This can be based on pre-programmed libraries or alternatively on real-time image-to-haptics conversation algorithms. Moreover, haptics allow the user a greater degree of control; the device can be explored at the discretion of the individual when they actively explore the tablet with their hand, leaving their mouth (and voice) unobstructed. These collective features may also augment the accessibility of such devices to both children and the elderly alike. However, we would also note that 4 of our 14 patients (i.e., 29%) did not meet our inclusion criterion of greater than chance levels with upright stimuli, independent of their angle of rotation. We can only speculate as to the potential causes and contributing factors. However, it is in our view unlikely that experience with Braille or the etiology of visual impairment has a direct link to performance with the haptic tablet. Both groups included individuals with either congenital or acquired impairments as well as individuals literate and illiterate in Braille. It will be important for further applications of this technology to establish if and how tactile sensitivity and discrimination abilities may underscore abilities to quickly learn to use this device. These points notwithstanding there are a number of promising domains for the application of this technology. One is the transmission of the concept of size-invariance (and perhaps also perspective invariance that can help promote both egocentric and allocentric representations) of haptic stimuli. This is an aspect that requires further exploration.

To date, devices including BrainPort and TDU have shown to be effective for functions ranging from object recognition, including measures of “visual” acuity (Chebat et al., 2007; Arnoldussen and Fletcher, 2012), to tasks requiring actions coordinated with mental representations of the perceived tactile objects (reviewed in Bach-y-Rita and Kercel, 2003). The present results similarly provide evidence that participants were able to understand the shape and even the form that the letters are haptically presented in (i.e., normal vs. mirror-reversed), speaks in favor of the application of digital haptics for simulation of spatial features. This is important, as spatial features rely on internal representations, and are directly related to spatial behaviors (Thinus-Blanc and Gaunet, 1997), such as object manipulation, localization, and spatial mapping. Ongoing work from our laboratory focuses on the applicability of this haptic technology in simulating trajectories modeled on a realistic indoor environment (i.e., an apartment's layout and corridors). Thus, the spatial functions that this technology has the potential to support can be directly extended to independent mobility.

The main innovation from our study is the successful application of a digital method. This is important, as digital rehabilitation methods promise to alleviate the medical field by reducing the necessary resources. Digital haptic stimulation finds applications not only in restoring spatial functions in the blind and visually-impaired, but also for rehabilitation of such functions in participants after sight restoration, for example in sight-restored cataract patients (McKyton et al., 2015). Specifically (McKyton et al., 2015), cataract operated children and young adults demonstrated immense deficits in mid-level visual processing (such as 3D object shapes) after cataract removal, despite intact low-level visual abilities. Using digital haptics, such patients may retrain their deficient spatial skills, in a safe, easy, and cost-effective way. As these representations have a “supra-modal” nature, digital tactile stimulation could aid existing therapy techniques to help patients encode a more abstract object representation. Thus, it is evident that the applicability of digital haptics is very promising for the medical domain.

## Conclusion

Participants with various forms of visual impairments or blindness were able to use a digital haptic technology to create mental representations of objects. It further suggests that these participants, unlike their sighted counterparts, seem to rely on a more conceptual encoding procedure that is not item-specific, thereby making more use of perceptual information, as well as of a possible multisensory working memory. Our results have important implications for rehabilitation regimes of object-related, spatial and mobility functions using sensory substitution, as well as for virtual simulated sensory perception methods more generally. Thus, our study highlights the merits of using innovative digital technologies as an application in rehabilitation.

## Data Availability Statement

The data that support the findings of this study are available from the corresponding author, MM, upon reasonable request.

## Ethics Statement

The studies involving human participants were reviewed and approved by Vaudois Cantontal Ethics Committee. The patients/participants provided their written informed consent to participate in this study.

## Author Contributions

RT and MM are responsible for the study concept and design. RT, FA, and JR acquired the data and worked with the participants. CC provided instrumentation and software. The analysis and interpretation of data were carried out by RT and MM. The manuscript was drafted by RT and MM. All authors provided input on revisions to the manuscript. All authors approved the final manuscript.

### Conflict of Interest

CC is the CEO and Founder of Hap2U. CC thus has commercial, proprietary, and financial interest in Hap2U, which provided the haptic tablet device instrument related to this article. The remaining authors declare that the research was conducted in the absence of any commercial or financial relationships that could be construed as a potential conflict of interest.

## References

[B1] Amedi A. Floel A. Knecht S. Zohary E. Cohen L. G. (2004). Transcranial magnetic stimulation of the occipital pole interferes with verbal processing in blind subjects. Nat. Neurosci. 7, 1266–1270. 10.1038/nn1328 15467719

[B2] Amedi A. Hofstetter S. Maidenbaum S. Heimler B. (2017). Task selectivity as a comprehensive principle for brain organization. Trends Cogn. Sci. 21, 307–310. 10.1016/j.tics.2017.03.007 28385460

[B3] Amedi A. Malach R. Hendler T. Peled S. Zohary E. (2001). Visuo-haptic object-related activation in the ventral visual pathway. Nat. Neurosci. 4, 324–330. 10.1038/85201 11224551

[B4] Arnoldussen A. Fletcher D. (2012). Visual perception for the blind: the brainport vision device. Retin. Physician 9, 32–34.

[B5] Auvray M. Hanneton S. O'Regan J. K. (2007). Learning to perceive with a visuo-auditory substitution system: localisation and object recognition with “The voice.” Perception 36, 416–430. 10.1068/p5631 17455756

[B6] Bach-y-Rita P. Danilov Y. Tyler M. Grimm R. (2005). Late human brain plasticity: vestibular substitution with a tongue brainport human-machine interface. Intellectica 40, 115–122. 10.3406/intel.2005.1362

[B7] Bach-y-Rita P. Kercel W. S. (2003). Sensory substitution and the human-machine interface. Trends Cogn. Sci. 7, 541–546. 10.1016/j.tics.2003.10.013 14643370

[B8] Bethell-fox C. E. Shepard R. N. (1988). Mental rotation : effects of stimulus complexity and familiarity. J. Exp. Psychol. 14, 12–23. 10.1037/0096-1523.14.1.12

[B9] Bonavolonta P. Travade I. Forte R. Rebeyrotte I. Adenis J.-P. Robert P.-Y. (2010). Intérêt de mesurer les acuités visuelles très basses dans un centre de réadaptation pour déficients visuels. J. Fr. Ophtalmol. 33, 391–396. 10.1016/j.jfo.2010.03.013 20493585

[B10] Carpenter P. A. Eisenberg P. (1978). Mental rotation and the frame of reference in blind and sighted individuals. Percept. Psychophys. 23, 117–124. 10.3758/BF03208291 643507

[B11] Chebat D. R. Rainville C. Kupers R. Ptito M. (2007). Tactile-'visual' acuity of the tongue in early blind individuals. Neuroreport 18, 1901–1904. 10.1097/WNR.0b013e3282f2a63 18007183

[B12] Collignon O. Dormal G. De Heering A. Lepore F. Lewis T. L. Maurer D. (2015). Long-lasting crossmodal cortical reorganization triggered by brief postnatal visual deprivation. Curr. Biol. 25, 2379–2383. 10.1016/j.cub.2015.07.036 26299512

[B13] Collignon O. Lassonde M. Lepore F. Bastien D. Veraart C. (2007). Functional cerebral reorganization for auditory spatial processing and auditory substitution of vision in early blind subjects. Cereb. Cortex 17, 457–465. 10.1093/cercor/bhj162 16581983

[B14] Collignon O. Renier L. Bruyer R. Tranduy D. Veraart C. (2006). Improved selective and divided spatial attention in early blind subjects. Brain Res. 1075, 175–182. 10.1016/j.brainres.2005.12.079 16460716

[B15] Collignon O. Voss P. Lassonde M. Lepore F. (2009). Cross-modal plasticity for the spatial processing of sounds in visually deprived subjects. Exp. Brain Res. 192:343. 10.1007/s00221-008-1553-z 18762928

[B16] Doane B. K. Mahatoo W. Heron W. Scott T. H. (1959). Changes in perceptual function after isolation. Can. J. Psychol. 13, 210–219. 10.1037/h0083772 13817038

[B17] Dwivedi A. N. Bali R. K. James A. E. Naguib R. Johnston D. (2002). Merger of knowledge management and information technology in healthcare : opportunities and challenges. Can. Conf. Electr. Comput. Eng. 2, 1194–1199. 10.1109/CCECE.2002.1013118

[B18] Facchini S. Aglioti S. M. (2003). Short term light deprivation increases tactile spatial acuity in humans. Neurology 60, 1998–1999. 10.1212/01.WNL.0000068026.15208.D0 12821752

[B19] Fary R. E. Briffa N. K. (2011). Monophasic electrical stimulation produces high rates of adverse skin reactions in healthy subjects. Physiother. Theory Pract. 27, 246–251. 10.3109/09593985.2010.487926 20690879

[B20] Field A. Miles J. Field Z. (2012). Discovering Statistics Using R. London, UK: Sage Publications.

[B21] Goldreich D. Kanics I. M. (2003). Tactile acuity is enhanced in blindness. J. Neurosci. 23, 3439–3445. 10.1523/JNEUROSCI.23-08-03439.2003 12716952 PMC6742312

[B22] Gori M. Cappagli G. Tonelli A. Baud-Bovy G. Finocchietti S. (2016). Devices for visually impaired people: high technological devices with low user acceptance and no adaptability for children. Neurosci. Biobehav. Rev. 69, 79–88. 10.1016/j.neubiorev.2016.06.043 27484870

[B23] Grant A. C. Thiagarajah M. C. Sathian K. (2000). Tactile perception in blind braille readers: a psychophysical study of acuity and hyperacuity using gratings and dot patterns. Percept. Psychophys. 62, 301–312. 10.3758/BF03205550 10723209

[B24] Hollins M. Risner S. R. (2000). Evidence for the duplex theory of tactile texture perception. Percept. Psychophys. 62, 695–705. 10.3758/BF03206916 10883578

[B25] Iachini T. Ruggiero G. Bartolo A. Rapuano M. Ruotolo F. (2019). The Effect of body-related stimuli on mental rotation in children, young and elderly adults. Sci. Rep. 9:1169. 10.1038/s41598-018-37729-7 30718610 PMC6362092

[B26] Jordan K. Heinze H. J. Lutz K. Kanowski M. Jäncke L. (2001). Cortical activations during the mental rotation of different visual objects. Neuroimage 13, 143–152. 10.1006/nimg.2000.0677 11133317

[B27] Kirkwood A. Rioult M. G. Bear M. F. (1996). Experience-dependent modification of synaptic plasticity in visual cortex. Nature 381, 526–528. 10.1038/381526a0 8632826

[B28] Kuyk T. Elliott J. L. Wesley J. Scilley K. McIntosh E. Mitchell S.. (2004). Mobility function in older veterans improves after blind rehabilitation. J. Rehabil. Res. Dev. 41, 337–346. 10.1682/JRRD.2003.03.0038 15543450

[B29] Lacey S. Sathian K. (2012). Representation of object form in vision and touch, in The Neural Bases of Multisensory Processes (Boca Raton, FL: CRC Press; Taylor & Francis), 179–187. 10.1201/b11092-13 22593875

[B30] Lacey S. Campbell C. (2006). Mental representation in visual/haptic crossmodal memory: evidence from interference effects. Q. J. Exp. Psychol. 59, 361–376. 10.1080/17470210500173232 16618639

[B31] Lacey S. Campbell C. Sathian K. (2007a). Vision and touch: multiple or multisensory representations of objects? Perception 36, 1513–1521. 10.1068/p5850 18265834

[B32] Lacey S. Pappas M. Kreps A. Lee K. Sathian K. (2009). Perceptual learning of view-independence in visuo-haptic object representations. Exp. Brain Res. 198, 329–337. 10.1007/s00221-009-1856-8 19484467 PMC2987670

[B33] Lacey S. Peters A. Sathian K. (2007b). Cross-modal object recognition is viewpoint-independent. PLoS ONE 2:e890. 10.1371/journal.pone.0000890 17849019 PMC1964535

[B34] Lahav O. Schloerb D. W. Srinivasan M. A. (2012). Newly blind persons using virtual environment system in a traditional orientation and mobility rehabilitation program: a case study. Disabil. Rehabil. Assist. Technol. 7, 420–435. 10.3109/17483107.2011.635327 22112148

[B35] Legge G. E. Madison C. Vaughn B. N. Cheong A. M. Y. Miller J. C. (2008). Retention of high tactile acuity throughout the life span in blindness. Percept. Psychophys. 70, 1471–1488. 10.3758/PP.70.8.1471 19064491 PMC3611958

[B36] Legood R. Scuffham P. Cryer C. (2002). Are we blind to injuries in the visually impaired? A review of the literature. Inj. Prev. 8, 155–160. 10.1136/ip.8.2.155 12120837 PMC1730864

[B37] Leon-Sarmiento F. E. Hernandez H. G. Schroeder N. (2008). Abnormal tactile discrimination and somatosensory plasticity in familial primary hyperhidrosis. Neurosci. Lett. 441, 332–334. 10.1016/j.neulet.2008.06.016 18577425

[B38] Manduchi R. Kurniawan S. (2011). Mobility-related accidents experienced by people with visual impairment. Insight Res. Pract. Vis. Impair. Blind. 4, 44–54. Available online at: http://users.soe.ucsc.edu/~manduchi/papers/MobilityAccidents.pdf

[B39] Marmor G. S. Zaback L. A. (1976). Mental rotation by the blind: does mental rotation depend on visual imagery? J. Exp. Psychol. Hum. Percept. Perform. 2, 515–521. 10.1037/0096-1523.2.4.515 1011000

[B40] Masson H. L. Bulthé J. De Beeck H. P. O. Wallraven C. (2016). Visual and haptic shape processing in the human brain: unisensory processing, multisensory convergence, and top-down influences. Cereb. Cortex 26, 3402–3412. 10.1093/cercor/bhv170 26223258

[B41] McKyton A. Ben-Zion I. Doron R. Zohary E. (2015). The limits of shape recognition following late emergence from blindness. Curr. Biol. 25, 2373–2378. 10.1016/j.cub.2015.06.040 26299519

[B42] Merabet L. B. Rizzo J. F. Amedi A. Somers D. C. Pascual-Leone A. (2005). What blindness can tell us about seeing again: merging neuroplasticity and neuroprostheses. Nat. Rev. Neurosci. 6, 71–77. 10.1038/nrn1586 15611728

[B43] Murray M. M. Lewkowicz D. J. Amedi A. Wallace M. T. (2016). Multisensory processes: a balancing act across the lifespan. Trends Neurosci. 39, 567–579. 10.1016/j.tins.2016.05.003 27282408 PMC4967384

[B44] Murray M. M. Matusz P. J. Amedi A. (2015). Neuroplasticity: unexpected consequences of early blindness. Curr. Biol. 25, R998–R1001. 10.1016/j.cub.2015.08.054 26485377

[B45] Murray M. M. Molholm S. Michel C. M. Heslenfeld D. J. Ritter W. Javitt D. C.. (2005). Grabbing your ear: rapid auditory-somatosensory multisensory interactions in low-level sensory cortices are not constrained by stimulus alignment. Cereb. Cortex 15, 963–974. 10.1093/cercor/bhh197 15537674

[B46] Noffsinger R. Chin S. (2000). Improving the delivery of care and reducing healthcare costs with the digitization of information. J. Healthc. Inf. Manag. 14, 23–30. 11066646

[B47] Pascual-Leone A. Amedi A. Fregni F. Merabet L. B. (2005). The plastic human brain cortex. Annu. Rev. Neurosci. 28, 377–401. 10.1146/annurev.neuro.27.070203.144216 16022601

[B48] Patel H. Congdon N. Strauss G. Lansingh C. (2017). A need for standardization in visual acuity measurement. Arq. Bras. Oftalmol. 80, 332–337. 10.5935/0004-2749.20170082 29160549

[B49] Peirce J. W. (2007). PsychoPy—psychophysics software in python. J. Neurosci. Methods 162, 8–13. 10.1016/j.jneumeth.2006.11.017 17254636 PMC2018741

[B50] Pietrini P. Furey M. L. Emiliano R. (2004). Além das imagens sensoriais: Representação baseada em objetos na via ventral humana. Proc. Natl. Acad. Sci. U.S.A. 101, 5658–5663. 10.1073/pnas.0400707101 15064396 PMC397466

[B51] Prather S. C. Sathian K. (2002). Mental rotation of tactile stimuli. Cogn. Brain Res. 14, 91–98. 10.1016/S0926-6410(02)00063-0 12063132

[B52] Prausnitz M. R. (1996). The effects of electric current applied to skin: a review for transdermal drug delivery. Adv. Drug Deliv. Rev. 18, 395–425. 10.1016/0169-409X(95)00081-H

[B53] Pribram K. H. (1971). Languages of the Brain: Experimental Paradoxes and Principles in Neuropsychology. Oxford: Prentice-Hall, 432.

[B54] Pring L. (1988). The ‘reverse-generation’ effect: a comparison of memory performance between blind and sighted children. Br. J. Psychol. 79, 387–400. 10.1111/j.2044-8295.1988.tb02297.x 3167500

[B55] Ptito M. Moesgaard S. M. Gjedde A. Kupers R. (2005). Cross-modal plasticity revealed by electrotactile stimulation of the tongue in the congenitally blind. Brain 128, 606–614. 10.1093/brain/awh380 15634727

[B56] R Core Team. (2018). R: a Language and Environment for Statistical Computing. Vienna: R Foundation for Statistical Computing. Available online at: https://www.R-project.org

[B57] Ratcliff R. (1993). Methods for dealing with reaction time outliers. Psychol. Bull. 114, 510–532. 10.1037/0033-2909.114.3.510 8272468

[B58] Rekik Y. Vezzoli E. Grisoni L. (2017). Understanding users' perception of simultaneous tactile textures, in MobileHCI'17 Proceedings of the 19th International Conference on Human-Computer Interaction With Mobile Devices and Services (Vienna: ACM), 229–238.

[B59] Röder B. Rösler F. (2003). Memory for environmental sounds in sighted, congenitally blind and late blind adults: evidence for cross-modal compensation. Int. J. Psychophysiol. 50, 27–39. 10.1016/S0167-8760(03)00122-3 14511834

[B60] Röder B. Rösler F. Hennighausen E. (1997). Different cortical activation patterns in blind and sighted humans during encoding and transformation of haptic images. Psychophysiology 34, 292–307. 10.1111/j.1469-8986.1997.tb02400.x 9175444

[B61] Rokem A. Ahissar M. (2009). Interactions of cognitive and auditory abilities in congenitally blind individuals. Neuropsychologia 47, 843–848. 10.1016/j.neuropsychologia.2008.12.017 19138693

[B62] Rovira K. Deschamps L. Baena-Gomez D. (2011). Mental rotation in blind and sighted adolescents: the effects of haptic strategies. Rev. Eur. Psychol. Appl. 61, 153–160. 10.1016/j.erap.2011.05.001

[B63] Sampaio E. Maris S. Bach-y-Rita P. (2001). Brain plasticity: ‘visual’ acuity of blind persons via the tongue. Brain Res. 908, 204–207. 10.1016/S0006-8993(01)02667-1 11454331

[B64] Sathian K. (2005). Visual cortical activity during tactile perception in the sighted and the visually deprived. Dev. Psychobiol. 46, 279–286. 10.1002/dev.20056 15772968

[B65] Sednaoui T. Vezzoli E. Dzidek B. Lemaire-Semail B. Chappaz C. Adams M. (2017). Friction reduction through ultrasonic vibration part 2: experimental evaluation of intermittent contact and squeeze film levitation. IEEE Trans. Haptics 10, 208–216. 10.1109/TOH.2017.2671376 28222001

[B66] Shepard R. N. Metzler J. (1971). Mental rotation of three-dimensional objects. Science 171, 701–703. 10.1126/science.171.3972.701 5540314

[B67] Stilla R. Sathian K. (2008). Selective visuo-haptic processing of shape and texture. Hum. Brain Mapp. 29, 1123–1138. 10.1002/hbm.20456 17924535 PMC3060058

[B68] Struiksma M. E. Noordzij M. L. Postma A. (2009). What is the link between language and spatial images? Behavioral and neural findings in blind and sighted individuals. Acta Psychol. 132, 145–156. 10.1016/j.actpsy.2009.04.002 19457462

[B69] Thinus-Blanc C. Gaunet F. (1997). Representation of space in blind persons: vision as a spatial sense? Psychol. Bull. 121, 20–42. 10.1037/0033-2909.121.1.20 9064698

[B70] Thomas M. Dalecki M. Abeln V. (2013). EEG coherence during mental rotation of letters, hands and scenes. Int. J. Psychophysiol. 89, 128–135. 10.1016/j.ijpsycho.2013.06.014 23797146

[B71] Tivadar R. I. Rouillard T. Chappaz C. Knebel J.-F. Turoman N. Anaflous F.. (2019). Mental rotation of digitally-rendered haptic objects. Front. Integr. Neurosci. 13:7. 10.3389/fnint.2019.00007 30930756 PMC6427928

[B72] Vezzoli E. Sednaoui T. Amberg M. Giraud F. Lemaire-Semail B. (2016). Texture rendering strategies with a high fidelity-capacitive visualhaptic friction control device, in Proceedings, Part I, of the 10th International Conference on Human Haptic: Sensing and Touch Enabled Computer Applications (Cham: Springer), 251–260.

[B73] Vezzoli E. Vidrih Z. Giamundo V. Lemaire-Semail B. Giraud F. Rodic T.. (2017). Friction reduction through ultrasonic vibration part 1: modelling intermittent contact. IEEE Trans. Haptics 10, 196–207. 10.1109/toh.2017.2671432 28222002

[B74] Vinter A. Fernandes V. Orlandi O. Morgan P. (2012). Exploratory procedures of tactile images in visually impaired and blindfolded sighted children: how they relate to their consequent performance in drawing. Res. Dev. Disabil. 33, 1819–1831. 10.1016/j.ridd.2012.05.001 22699255

[B75] Wan C. Y. Wood A. G. Reutens D. C. Wilson S. J. (2010). Congenital blindness leads to enhanced vibrotactile perception. Neuropsychologia 48, 631–635. 10.1016/j.neuropsychologia.2009.10.001 19819246

[B76] White M. J. (1980). Naming and categorization of tilted alphanumeric characters do not require mental rotation. Bull. Psychon. Soc. 15, 153–156. 10.3758/BF03334494

[B77] Wong M. Gnanakumaran V. Goldreich D. (2011). Tactile spatial acuity enhancement in blindness: evidence for experience-dependent mechanisms. J. Neurosci. 31, 7028–7037. 10.1523/JNEUROSCI.6461-10.2011 21562264 PMC6703211

[B78] World Health Organization (2000). Blindness: Vision 2020-The Global Initiative for the Elimination of Avoidable Blindness, Fact Sheet 213. Available online at: https://www.who.int/mediacentre/factsheets/fs213/en/

[B79] Zubek J. P. Flye J. Willows D. (1964). Changes in cutaneous sensitivity after prolonged exposure to unpatterned light. Psychon. Sci. 1, 283–284. 10.3758/BF03342913

